# Criterion Validity of a Short Food Frequency Questionnaire for Mexican American Adults

**DOI:** 10.3390/nu14235075

**Published:** 2022-11-29

**Authors:** Natalia I. Heredia, Deanna M. Hoelscher, Vanessa Vega, Belinda M. Reininger

**Affiliations:** 1 Department of Health Promotion and Behavioral Sciences, School of Public Health, The University of Texas Health Science Center at Houston (UTHealth), Houston, TX 77030, USA; 2 Michael and Susan Dell Center for Health Living, UTHealth School of Public Health, Austin, TX 78701, USA; 3 M Health Fairview Masonic Children’s Hospital, Minneapolis, MN 55454, USA; 4 Brownsville Regional Campus, UTHealth School of Public Health, Brownsville, TX 78520, USA

**Keywords:** dietary survey, Mexican American, Hispanic, validation, food frequency questionnaire, 24 h recall

## Abstract

Background: The purpose of this study was to validate the School Physical Activity and Nutrition (SPAN) Food Frequency Questionnaire (FFQ) for Mexican American adults (SPAN MAA). Methods: A sample of 100 Mexican American adult participants was drawn from the Cameron County Hispanic Cohort (on the Texas–Mexico border). We used Spearman rank order correlation coefficients, kappa statistics, and percent agreement to compare the SPAN MAA questionnaire to a 24 h recall collected on the same day. Results: Of 100 participants, 93 were included in the analyses. One item showed substantial (>0.6), five items moderate (>0.4), five items fair (>0.2) and three items little to no agreement (<0.2). Items with low agreement were those reported in low frequencies by study participants. Conclusions: SPAN MAA FFQ had moderate to fair agreement between instruments across both sexes, making this brief questionnaire a useful tool to quickly assess the dietary intake patterns of Mexican American adults.

## 1. Introduction

Excess calorie consumption and poor dietary habits are chief factors influencing the onset of obesity, a public health concern worldwide [1,2,3]. Poor dietary habits also contribute to chronic diseases, including cardiovascular disease [4], cancer [5], diabetes [6], and non-alcoholic fatty liver disease [7]. Along the Texas–Mexico border, the prevalence of obesity is at epidemic proportions, with an estimated 50.9% of the population classified as having obesity [8]. To effectively intervene to improve dietary habits, it is first necessary to accurately measure the dietary patterns of the population, which is almost 90% Hispanic/Latino [9], and mostly Mexican American along the Texas–Mexico border.

Food frequency questionnaires (FFQ) are an epidemiological tool for assessing dietary habits that are easy to use and relatively cost-effective, making them popular surveillance tools [10,11]. Though FFQs are widely used as epidemiologic dietary surveys, differences in food supply and dietary habits from one population to another make it necessary for a FFQ tool to be tested each time it is introduced in a new population [12,13]. While a few FFQs have been tested among Latin American populations such as Cuban Americans [14] and Caribbean Latinos [15], there remains a need for a brief FFQ, adapted to include the linguistic and cultural differences specific to Mexican American adults in a border population.

One FFQ that has been used to measure dietary intake among Mexican American is the School Physical Activity and Nutrition (SPAN) FFQ. This questionnaire was originally developed for use with school-aged children in Texas to determine mean group intakes of select foods and meal/snack patterns that may underlie obesity [16,17,18]. Cognitive testing was used during the development of the SPAN to examine and further refine items to increase participant comprehension [16] It has also been tested for readability, cultural relevance, reproducibility, and validity among African American and Mexican American children and adolescents [16,19]. Given the low-literacy levels of the adult population on the Texas–Mexico border [20], this type of FFQ could be useful for surveillance in this population.

Thus, this study aims to evaluate a brief dietary survey, the SPAN for Mexican American Adults (SPAN MAA), by comparing its responses to that of a 24 h recall conducted during the same one-day time period. It is hypothesized that the specific food categories on the SPAN MAA will be positively associated with food intake as captured by 24 h recalls in a Mexican American adult population.

## 2. Methods

### 2.1. Study Design and Participants

This study was conducted with participants from the Cameron County Hispanic Cohort (CCHC) [21], a cohort of Mexican Americans from a randomly selected, household sample in Cameron County, Texas, located along the US–Mexico border [21,22]. Participants ages 18 to 67 years, who spoke English or Spanish as their primary language were eligible for enrollment in the study. Both new recruits to the CCHC as well as previously enrolled CCHC participants returning for their five-year follow-up visit were recruited. Of the 100 CCHC participants approached for recruitment into the study, all agreed to participate. This study’s human subject protections and all associated documents were approved by the Institutional Review Board at the University of Texas Health Science Center at Houston.

Participants completed informed consent. All consents, questionnaires, and other study steps were conducted in the participants’ reported preferred language (either English or Spanish). Participants were randomly assigned to either group A or group B. The assignment process used a simple randomization technique of drawing a card from a stack indicating group assignment. Group A began with the SPAN MAA, followed by participation in the CCHC clinic visit, and finished with the 24 h recall. Group B began with the 24 h recall, followed by the CCHC clinic visit, and then the SPAN MAA. The process allowed for at least two hours to elapse between the administration of the SPAN MAA and the 24 h recall. Two groups with a separate order of administrative for the 24 h recall and the SPAN MAA questionnaire were used to control for a potential order effect [19].

The SPAN MAA and 24 h recall were completed during weekdays, between Tuesday and Friday. Previous studies have found that eating patterns differ on weekdays and weekends and because of funding limitations, this study focused on weekday eating habits [19]. Both the SPAN MAA and the 24 h food recall took approximately 20 to 45 min each to administer and were completed using an interview format in which a trained data collector read each question out loud to the participant in the participant’s language of choice and recorded the responses. Both instruments assessed the same time period (yesterday’s food intake).

The SPAN MAA was also administered orally with the trained data collector reading the directions of the questionnaire aloud to the participant and then reading each item and hand-recording the participant’s answers. Based on food intake from the previous day, participants selected from the following answer choices: No, I did not eat any of the foods listed above yesterday (=0); Yes, I ate one of these foods 1 time yesterday (=1); Yes, I ate one of these foods 2 times yesterday (=2); and Yes, I ate one of these foods three or more times yesterday (=3).

The 24 h recall administration protocol followed the study protocol used in the original SPAN validity study [19]. A trained data collector administered a 24 h multiple-pass food recall. For each participant, the data collector manually recorded the previous day’s food intake, followed by a review of the food items, recording quantity consumed by probing for serving size based on food models, asking for more detail about each food consumed such as additions, and finally concluding the recall with a review of items mentioned and quantity consumed [19]. After each session, the data collector completed a handwritten food intake form (FIF), which was later entered into an Excel file detailing the information collected during the survey [19]. These forms were processed using the Food Intake Analysis System (FIAS) using standard protocols.

### 2.2. Measures

***SPAN.*** The SPAN MAA is a brief dietary survey modeled after the 4th grade version of the SPAN FFQ [16]. This version includes 20 items (Table 1), covering healthy foods groups (vegetables, fruits, fruit juice, whole grains, healthy meats, and beans) and unhealthy foods groups (unhealthy meats, frozen desserts, candy, baked goods, French fries/chips, non-whole grains, sugar-sweetened beverages, and diet soft drinks). Respondents are asked how many times they ate a particular food the previous day, with response options from 0 to 3 or more times.

The SPAN FFQ for fourth grade students has a readability level of grade 4.98, as determined by the Dale–Chall formula [16]. This version of SPAN FFQ was chosen as the foundation of SPAN MAA because its readability level best met the needs of the large low-literacy population living in the Texas–Mexico border area [20]. However, adaptations were made to the original SPAN FFQ to ensure that it was appropriate for the priority population of adult Mexican American residents living along the US–Mexico border. At four different meetings with community members, the community and bilingual staff reviewed and discussed the linguistic and cultural appropriateness of the measure, including food options and wording of items. For example, various questions were altered to include traditional Mexican food examples, such as tortillas (non-whole grains) and chorizo, a Mexican pork sausage (unhealthy meats). SPAN MAA was also pilot tested with community health workers who gave feedback on wording, translation, food options, question sequence order, and skip patterns. The entire instrument underwent forward and backward translation, and was updated to address the comments and suggestions received. While the original SPAN FFQ was designed to be self-administered, recommendations emerged during the adaptation process for this measure to be verbally administered by staff members.

All responses to questions within a food group were summed to form a composite score [23,24].

#### 2.2.1. 24 h Recall

The 24 h recall assesses all foods and drinks consumed within the past 24 h. The 24 h recall is an appropriate measurement tool for assessing dietary intake in low-literacy populations because participants are not necessarily required to read or write to complete the recall, as long as it is interviewer-administered [25,26].

Foods on the 24 h recalls were entered into the Food Intake Analysis System (FIAS) and classified initially into MyPlate food group categories. These food groups were then recoded into food groups consistent with categories on the SPAN MAA (e.g., breads, rolls, tortillas, fried meats, etc.) This allowed a comparison between the foods from the 24 h recalls and the food category questions on the SPAN MAA instrument using kappa statistic and percent agreement calculations.

#### 2.2.2. Covariates and Other Variables

Demographic variables self-reported by the participant included sex and age. Height was measured using a stadiometer to the nearest 0.1 cm. Weight was measured with a calibrated beam balance and was recorded at the nearest 0.1 kg. Body Mass Index (BMI) was calculated by dividing weight by height in meters squared [27] and cut points for underweight, healthy weight, overweight, obesity, and severe obesity were based on widely accepted standards [28].

### 2.3. Data Analysis

Means, standard deviations and percentages were calculated for demographic variables. We calculated Pearson’s chi-squared or Fischer’s exact test to compare SPAN MAA and 24 h recall responses. The highest-level category (i.e., 3 or more times a day.) was collapsed when the cells were very small. We calculated Spearman rank order correlation coefficients, Cicchetti-Allison weighted kappa and percent agreement of the food items between the SPAN MAA and the 24 h recall for the overall sample and by both sex and age. A kappa coefficient ranges from 0 to 1, with 1 indicating perfect agreement. Weighted kappa coefficients are used for ordinal scales [29]. All data were analyzed at the aggregate level, which is appropriate for “yesterday” type items [16]. Total caloric intake based on 24 h Recall was calculated using FIAS for each participant. Additionally, minimum daily calorie levels were determined using the MyPyramid Food Intake Pattern Calorie Levels for each participant based on age and using the sedentary lifestyle minimum daily caloric intake requirements. Study participants who reported consuming half or less of the recommended daily calorie levels were excluded from analysis (n = 6), though two other participants who reported consuming double or more of the recommended daily calories were retained because both reported a feasible diet and caloric intake. Based on these exclusions, the analysis for this study included 94 participants. No participants were classified as underweight, so that category was omitted from analyses. Data were analyzed using SPSS 17.0 (SPSS Inc., Chicago, IL, USA, released 2008) and SAS 9.4 (SAS Institute Inc., Cary, NC, USA released 2013).

## 3. Results

Of the 94 participants included in analysis for this study, all were Mexican American, and the majority (66%) were female and had either overweight, obesity or severe obesity (85%) (Table 2).

We compared the frequency of foods as reported on the SPAN MAA and the 24 h recall by category. The results indicate that there are statistically significant differences between responses for fruit, fruit juice, whole grains, healthy meats, beans, unhealthy meats, frozen desserts, candy, fries/chips, baked goods, sugar sweetened beverages, and diet soft drinks (Table 3).

Ten of the categories had a percent agreement between the two measures over 56%, and five items had a percent agreement over 74.5% (Table 4). Overall, most dietary intake categories showed fair (Kappa 0.21 to 0.40) to moderate (Kappa 0.41–0.60) agreement between the SPAN MAA and the 24 h recall for the full sample (Table 4). We found substantial agreement (Kappa 0.61–0.80) between instruments in the full sample for diet soft drinks, and moderate agreement (Kappa 0.41–0.60) for fruit juice, healthy meats, beans, fries/chips, and sugar-sweetened beverages. There was fair agreement (Kappa between 0.21 and 0.4) between instruments in the full sample for fruit, unhealthy meats, frozen desserts, candy, and baked goods. Among women, we also found a moderate agreement between instruments for baked goods and fair agreement for vegetables, and among men, we found substantial agreement for fruit juice (Table 5). Among adults 46 years of age and older, we found substantial agreement for fries/chips and moderate agreement for baked goods (Table 6). We also found substantial agreement for sugar-sweetened beverages among adults 18–45 years of age. 

## 4. Discussion

This study aims to evaluate a brief dietary survey, SPAN MAA, by comparing its responses to that of a 24 h recall conducted during the same one-day time period. It was hypothesized that the specific food categories on the SPAN MAA would be positively associated with food intake as captured by 24 h recalls in a Mexican American adult population. We found that five of the food categories on the SPAN MAA had over 70% agreement with the same categories on the 24 h recall, and most categories showed a fair to moderate agreement as assessed by weighted Kappa, levels of agreement similar to other studies [16,19,30]. Food items that were easier to distinguish, eaten/drank on their own or narrowly defined, such as diet soft drinks (Kappa = 0.67), fries/chips (Kappa = 0.52), sugar-sweetened beverages (Kappa = 0.48), beans (Kappa = 0.48), healthy (i.e., baked) meats (Kappa = 0.44), and fruit juice (Kappa = 0.44), had substantial to moderate agreement between the two measures.

Several dietary intake categories showed poor agreement and will need further development for use with Mexican American adults. The difference in approach between the two dietary assessment methods may have contributed to the low agreement on some items; while SPAN MAA asked individuals to report food consumption by broad categories, the 24 h asked about each meal and then specifically examined all ingredients, helping individuals to more precisely recall their food consumption. This difference in approach may be why food items found in mixed dishes had low agreement. For example, vegetables, which participants may not recall consuming on the SPAN MAA if they were one of many ingredients in a taco or stew, had a Kappa of 0.17. Few participants reported consuming a side serving of vegetables, with responses on the 24 h recall indicating that the majority of vegetables were parts of mixed dishes. The purposeful simplicity of SPAN MAA questions, therefore, may have made it harder for individuals to recall and report the contents of mixed dishes with more complicated ingredients. In comparison, the prompting approach used during the 24 h recall may have supported participants in more accurately reporting these foods consumed as part of mixed dishes. While more precise recall is a positive attribute of the 24 h recall, the brevity of the SPAN MAA makes it more practical for use in our low-literacy population. For the vegetable item in particular, adding an additional prompt to think about vegetables consumed in mixed dishes may improve the agreement between the two instruments with minimal additional participant burden.

Differences in available response options between the two instruments may also contribute to low agreement, especially for certain items. The SPAN MAA asked for the number of times an item was consumed in a day, while the 24 h recall recorded number of servings. Participants may not have been aware of the appropriate serving size and either under or overreported their food intake. We can use sugar sweetened beverages as an example, though it did have moderate agreement between the two instruments. If a participant consumed one 32-ounce sugar sweetened beverage yesterday, it is likely on the SPAN MAA they would report a diet soft drink was consumed only one time. On the 24 h recall, this same consumption would have been recorded as several servings. The different response options could have contributed to lower levels of agreement.

Foods that were difficult to define also had poor agreement. For example, participants might have had difficulty differentiating between whole grains and non-whole grains. Whole grain foods had a Kappa of 0.06 and a percentage agreement of 47%, and non-whole grains had a Kappa of 0.02 and a percent agreement of 20.2%. This low agreement may be because on the SPAN MAA, participants had to differentiate between whole grains and refined grain products, with only broad descriptions of either category to assist them (see Table 1). This may in turn have caused confusion between different types of grain, confusion that may be lessened with more specific probing about the grain source of the product, which is a part of the 24 h recall protocol. Some additional clarification on the items that make up these two SPAN MAA categories may improve agreement between the two instruments.

Lastly, many food groups that showed good percent agreement but lower kappa values were foods consumed less frequently by participants. For example, the distribution of frozen desserts was very skewed, with very few participants on either assessment indicating that they consumed this food. However, despite a very high percent agreement (85%), the kappa was only 0.21, showing the impact that a skewed distribution has on kappa coefficients [31]. Similarly, while 51% of participants reported consuming one or more whole grains on the SPAN MAA, 84% reported consuming less than half a serving of whole grains on the 24 h recall. This skewed distribution on both instruments may have impacted the Kappa, which for whole grains was only 0.06.

The SPAN MAA survey was designed to assess dietary intake among a Hispanic/Latino adult population, so the primary validation outcomes were centered on the total study population; however, we also saw some preliminary differences in agreement between SPAN MAA and the 24 h recall by sex and age. We saw similar percent agreement but lower kappa among men compared to women for vegetables, candy and frozen desserts, indicating that limited consumption of these items among men led to skewed distributions and thus lower kappa coefficients. We saw the reverse for sugar sweetened beverages and diet soft drinks, that is, similar percent agreement in the context of lower kappa among women but not men. We also found much lower percent agreement in fruit juice and whole grains for women compared to men, and much higher agreement for fries/chips and baked goods for women compared to men, indicating potential sex-differences in the accurate reporting of certain items on either instrument. We also found differences in agreement by age, with greater percent agreement and kappa scores for vegetables, fries/chips and baked goods in older adults (≥46 years of age) as compared with adults 18 to 45 years of age, whereas most other items had similar scores or adults 18–45 years of age had higher weighted kappa coefficients and percent agreement than older adults. Since our study was not powered to examine differences by gender or age, and our stratified sample sizes were small, further work should be done to determine differences by demographic factors.

There are a few limitations of our study that need to be noted. The SPAN MAA, a brief survey, assessed important foods groups like fruit, vegetable, meat, grain, beans, sweets and soft drink consumption but generally is a limited dietary intake questionnaire. For example, it does not assess calcium intake through milk consumption, cheese and other dairy products, which provide important nutrients. Since the SPAN MAA is a brief dietary survey, our evaluation was limited to comparing the food groups present in the survey, and the instrument does not allow for us to assess agreement for total energy intake or dietary indices that require more expansive information. Our study used self-reported measures, which can create bias, especially among females, individuals with higher BMIs, those from a lower socioeconomic status or with less education and those concerned with body image or size, all of whom tend to underreport dietary intake [32,33]. However, our study was designed to compare one form of self-reported dietary assessment method against another, using 24 h recalls, which are the gold standard in dietary validation studies. Additionally, this study only compared weekday dietary intake and the criterion validity results may or may not apply to weekend dietary intake, which is a limitation. This study had many strengths. First, this study used an instrument that was previously evaluated in an ethnically diverse, elementary-school age population [19,34] and was adapted so that it was culturally appropriate to use with Mexican American adults with low literacy. We used three of the most common statistical tests for FFQ evaluation studies [19,30], and moreover, multiple statistical tests allow for a more complete and nuanced understanding of the study results [30]. While this tool should be evaluated in a larger population to reach more conclusive results, the sample size was comparable to that of other evaluation studies among adult populations [35,36,37]. Additionally, the possibility of survey bias due to order of administration was minimized through this study design by designating half of the study population to begin with the SPAN MAA followed by the 24 h food recall, while the other half began with the 24 h food recall followed by the SPAN MAA. The two hours between questionnaire administrations may have minimized the potential for reporting a remembered response [19]. Nevertheless, future improvements for the questionnaire are recommended, and will require further evaluation in a larger population.

## 5. Conclusions

The SPAN MAA FFQ showed fair to moderate agreement when compared to a 24 h recall and may be a useful tool to analyze dietary intake among Mexican American adults.

## Figures and Tables

**Table 1:** School Physical Activity and Nutrition for Mexican American Adults (SPAN MAA).

Healthy Food Groups
**Vegetables**
Yesterday, did you eat any orange vegetables like carrots, squash, or sweet potatoes?
Yesterday, did you eat a salad made with lettuce or any green vegetables like spinach, green beans, broccoli, or other greens?
Yesterday, did you eat any other vegetables like peppers, tomatoes, zucchini, asparagus, cabbage, cauliflower, cucumbers, mushrooms, eggplant, celery, or artichokes?
**Fruit**
Yesterday, did you eat any fruit? DO NOT COUNT fruit juice.
**Fruit Juice**
Yesterday, did you drink fruit juice? Fruit juice is a drink, which is over 100% juice, like orange juice, apple juice, or grape juice. DO NOT COUNT punch, Kool-Aid^®^, sports drinks, or other fruit-flavored drinks.
**Whole Grains**
Yesterday, did you eat any WHOLE WHEAT bread, buns, bagels, tortillas, or rolls?
Yesterday, did you eat any hot or cold WHOLE GRAIN cereal such as Special K^®^, Total^®^, Wheaties^®^, or Raisin Bran^®^?
**Healthy Meats**
Yesterday, did you eat any baked, grilled, broiled, or steamed fish or chicken? DO NOT COUNT fried chicken, fried fish, or fish sticks.
**Beans**
Yesterday, did you eat beans such as pinto beans, baked beans, kidney beans, refried beans, or pork & beans? DO NOT COUNT green beans.
**Unhealthy Food Groups**
**Unhealthy Meats**
Yesterday, did you eat hamburger meat, hot dogs, sausage (chorizo), steak, bacon or ribs?
Yesterday, did you eat any fried meat with a crust, like fried chicken, chicken nuggets, chicken fried steak, fried pork chops, fried fish, fish sticks, or milanesa?
**Frozen Desserts**
Yesterday, did you eat a frozen dessert? A frozen dessert is a cold, sweet food like ice cream, frozen yogurt, an ice cream bar.
**Candy**
Yesterday, did you eat any chocolate candy? DO NOT COUNT brownies or chocolate cookies.
Yesterday, did you eat any candy other than chocolate candy? COUNT hard, chewy or gummy candy. Do not count gum.
**Baked Goods**
Yesterday, did you eat sweet rolls, doughnuts, cookies, brownies, pies, or cake?
**Fries/Chips**
Yesterday, did you eat french fries or chips? Chips are potato chips, tortilla chips, Cheetos^®^, corn chips, or other snack chips.
**Non-Whole Grains**
Yesterday, did you eat any WHITE bread, buns, bagels, tortillas, or rolls?
**Sugar Sweetened Beverages**
Yesterday, did you drink any punch, Kool-Aid^®^, sports drinks, or other fruit-flavored drinks? DO NOT COUNT fruit juice.
Yesterday, did you drink any REGULAR (not diet) sodas or soft drinks?
**Diet Soft Drinks**
Yesterday, did you drink any DIET sodas or soft drinks (not regular)?

**Table 2:** Demographic characteristics of participants (n = 94).

Characteristic	n (%)
**Sex**	
Male	32 (34.0)
Female	62 (66.0)
**Age**	
18–25 years	12 (12.8)
26–35 years	16 (17.0)
36–45 years	19 (20.2)
46–55 years	24 (25.5)
60–65 years	20 (21.3)
66 years and older	3 (3.2)
**BMI Category**	
Healthy Weight (18.5–24.9)	14 (14.9)
Overweight (25–29.9)	36 (38.3)
Obesity (30–39.9)	37 (39.4)
Severe Obesity (≥40)	7 (7.4)
**Primary Language**	
English	27 (28.7)
Spanish	67 (71.3)

**Table 3:** Comparison of reported food consumption by category as collected by the SPAN MAA and 24 h recall (n = 94).

SPAN MAA		24 h Recall		*p* Value
Response Options (Times Eaten Yesterday)	n (%)	Range	n (%)	
**Healthy Food Groups**				
*Vegetables*				
0	40 (42.5)	0 ≤ x < 0.5	30 (31.9)	0.26
1	26 (27.7)	0.5 ≤ x < 1.5	46 (48.9)	
≥2	28 (29.8)	≥1.5	18 (19.2)	
*Fruit*				
0	56 (59.6)	0 ≤ x < 0.5	64 (68.1)	<0.001
1	28 (29.8)	0.5 ≤ x < 1.5	22 (23.4)	
≥2	10 (10.6)	≥1.5	8 (8.5)	
*Fruit Juice*				
0	71 (75.5)	0 ≤ x < 0.5	74 (78.7)	<0.001
1	17 (18.1)	0.5 ≤ x < 1.5	7 (7.5)	
≥2	6 (6.4)	≥1.5	13 (13.8)	
*Whole Grains*				
0	46 (48.9)	0 ≤ x < 0.5	79 (84.0)	<0.01
1	28 (29.8)	0.5 ≤ x < 1.5	10 (10.6)	
≥2	20 (21.3)	≥1.5	5 (5.3)	
*Healthy Meats*				
0	61 (64.9)	0 ≤ x < 0.5	67 (71.3)	<0.001
1	30 (31.9)	0.5 ≤ x < 1.5	11 (11.7)	
≥2	3 (3.2)	≥1.5	16 (17.0)	
*Beans*				
0	50 (53.2)	0 ≤ x < 0.5	62 (66.0)	<0.001
1	34 (36.2)	0.5 ≤ x < 1.5	16 (17.0)	
≥2	10 (10.6)	≥1.5	16 (17.0)	
**Unhealthy Food Groups**				
*Unhealthy Meats*				
0	38 (40.4)	0 ≤ x < 0.5	31 (33.0)	<0.01
1	39 (41.5)	0.5 ≤ x < 1.5	33 (35.1)	
≥2	17 (18.1)	≥1.5	30 (31.9)	
*Frozen Desserts*				
0	80 (85.1)	0	88 (94.7)	<0.01
≥1	14 (14.9)	≥1	5 (5.3)	
*Candy*				
0	73 (77.7)	0 ≤ x < 0.5	85 (90.4)	<0.01
1	21 (22.3)	≥0.5 ≤ x	9 (9.6)	
*Fries/Chips*				
0	60 (63.8)	0 ≤ x < 0.5	61 (64.9)	<0.001
1	31 (33.0)	0.5 ≤ x < 1.5	21 (22.3)	
≥2	3 (3.2)	≥1.5	12 (12.8)	
*Non-Whole Grains*				
0	45 (47.9)	0 ≤ x < 0.5	1 (1.1)	0.75
1	32 (34.0)	0.5 ≤ x < 1.5	3 (3.2)	
≥2	17 (18.1)	≥1.5	90 (95.7)	
*Baked Goods*				
0	63 (67.0)	0 ≤ x < 0.5	54 (57.5)	<0.001
1	23 (24.5)	0.5 ≤ x < 1.5	5 (5.3)	
≥2	8 (8.5)	≥1.5	35 (37.2)	
*Sugar Sweetened Beverages*				
0	35 (37.2)	0 ≤ x < 0.5	45 (47.9)	<0.001
1	29 (30.8)	0.5 ≤ x < 1.5	25 (26.6)	
2	30 (32.0)	≥1.5	24 (25.5)	
*Diet Soft Drinks*				
0	82 (87.2)	0 ≤ x < 0.5	86 (91.5)	<0.001
≥1	12 (12.8)	≥0.5	8 (8.5)	

**Table 4:** Comparison of SPAN MAA and 24 h recall (n = 94).

	Spearman Correlation (*p*)	Weighted Kappa Coefficient (95% CI, *p*)	Percent Agreement (%)
**Healthy Food Groups**			
Vegetables	0.21 (0.044)	0.17 (0.02–0.33, 0.0257)	42.6
Fruit	0.43 (<0.0001)	0.33 (0.18–0.49, 0.0001)	64.9
Fruit juice	0.65 (<0.0001)	0.44 (0.3–0.59, <0.0001)	74.5
Whole grains	0.31 (0.0025)	0.06 (−0.04–0.16, 0.2997)	46.8
Healthy meats	0.55 (<0.0001)	0.44 (0.29–0.59, <0.0001)	68.1
Beans	0.61 (<0.0001)	0.48 (0.34–0.61, <0.0001)	64.9
**Unhealthy Food Groups**			
Unhealthy meats	0.5 (<0.0001)	0.29 (0.16–0.42, <0.0001)	43.6
Frozen desserts ^a^	0.42 (<0.0001)	0.21 (−0.03–0.45, 0.0105)	85.1
Candy ^a^	0.35 (0.0006)	0.31 (0.08–0.54, 0.0008)	80.9
Fries/Chips ^a^	0.71 (<0.0001)	0.52 (0.37–0.66, <0.0001)	75.5
Non-Whole grains	0.13 (0.2241)	0.02 (0–0.05, 0.2086)	20.2
Baked goods	0.57 (<0.0001)	0.34 (0.21–0.47, <0.0001)	57.4
Sugar Sweetened Beverages	0.65 (<0.0001)	0.48 (0.35–0.61, <0.0001)	56.4
Diet Soft Drink s ^a^	0.68 (<0.0001)	0.67 (0.42–0.91, <0.0001)	93.6

^a^ Simple Kappa Coefficient for nominal scale.

**Table 5:** Comparison of SPAN MAA and 24 h recall, by sex (n = 94).

	Females (n = 62)		Males (n = 32)	
	Weighted Kappa Coefficient (95% CI, *p*)	Percent Agreement (%)	Weighted Kappa Coefficient (95% CI, *p*)	Percent Agreement (%)
**Healthy Food Groups**				
Vegetables	0.22 (0.04–0.41, 0.0162)	43.5	0.07 (−0.21–0.36, 0.5849)	40.6
Fruit	0.33 (0.13–0.52, 0.0013)	64.5	0.35 (0.1–0.6, 0.0152)	65.6
Fruit juice	0.38 (0.21–0.55, 0.0003)	66.1	0.64 (0.42–0.85, <0.0001)	90.6
Whole grains	0.06 (−0.04–0.17, 0.3595)	40.3	0.03 (−0.19–0.24, 0.8099)	59.4
Healthy meats	0.43 (0.23–0.62, <0.0001)	71	0.44 (0.21–0.68, 0.0006)	62.5
Beans	0.46 (0.27–0.65, <0.0001)	69.4	0.47 (0.27–0.67, 0.0003)	56.3
**Unhealthy Food Groups**				
Unhealthy meats	0.29 (0.13–0.45, 0.0005)	46.8	0.32 (0.13–0.51, 0.0043)	37.5
Frozen desserts ^a^	0.25 (−0.07–0.57, 0.0147)	85.5	0.11 (−0.15–0.37, 0.3843)	84.4
Candy ^a^	0.38 (0.1–0.67, 0.001)	82.3	0.14 (−0.21–0.49, 0.3204)	78.1
Fries/Chips ^a^	0.57 (0.37–0.77, <0.0001)	82.3	0.42 (0.19–0.64, 0.0004)	62.5
Non-Whole grains	0.04 (−0.01–0.08, 0.174)	24.2	0 (0–0)	12.5
Baked goods	0.43 (0.27–0.6, <0.0001)	61.3	0.17 (0.01–0.32, 0.0891)	50
SSB	0.43 (0.26–0.6, <0.0001)	54.8	0.57 (0.37–0.76, 0.0001)	59.4
Diet Soft Drinks ^a^	0.57 (0.2–0.94, <0.0001)	93.5	0.76 (0.45–1, <0.0001)	93.8

^a^ Simple Kappa Coefficient for nominal scale.

**Table 6:** Comparison of SPAN MAA and 24 h recall, by age (n = 94).

	18 to 45 (n = 47)		46 and Older (n = 47)	
	Weighted Kappa Coefficient (95% CI, *p*)	Percent Agreement (%)	Weighted Kappa Coefficient (95% CI, *p*)	Percent Agreement (%)
**Healthy Food Groups**				
Vegetables	0.05 (−0.16–0.25, 0.6779)	38.3	0.29 (0.08–0.51, 0.0064)	46.8
Fruit	0.36 (0.14–0.58, 0.0023)	72.3	0.29 (0.07–0.51, 0.0132)	57.4
Fruit juice	0.51 (0.39–0.62, <0.0001)	74.5	0.40 (0.15–0.66, 0.0007)	74.5
Whole grains	0.06 (−0.08–0.19, 0.4937)	44.7	0.06 (−0.08–0.21, 0.4249)	48.9
Healthy meats	0.53 (0.33–0.73, <0.0001)	72.3	0.35 (0.13–0.57, 0.0011)	63.8
Beans	0.53 (0.35–0.71, <0.0001)	68.1	0.42 (0.21–0.62, 0.0002)	61.7
**Unhealthy Food Groups**				
Unhealthy meats	0.26 (0.09–0.43, 0.0067)	38.3	0.32 (0.12–0.51, 0.0021)	48.9
Frozen desserts	0.19 (−0.13–0.52, 0.0256)	85.1	0.30 (−0.04–0.64, 0.0196)	85.1
Candy ^a^	0.22 (−0.12–0.55, 0.1011)	80.9	0.38 (0.07–0.68, 0.0031)	80.9
Fries/Chips	0.49 (0.32–0.67, <0.0001)	66	0.69 (0.48–0.89, <0.0001)	85.1
Non-Whole grains	0.03 (−0.03–0.08, 0.3182)	25.5	0.01 (0–0.03, 0.4935)	14.9
Baked goods	0.19 (0.02–0.36, 0.044)	48.9	0.50 (0.31–0.68, <0.0001)	66
SSB	0.61 (0.44–0.78, <0.0001)	66	0.31 (0.14–0.49, 0.0054)	46.8
Diet Soft Drinks ^a^	0.48 (−0.14–1, 0.0011)	95.7	0.70 (0.44–0.97, <0.0001)	91.5

^a^ Simple Kappa Coefficient for nominal scale.

## Data Availability

The data is available upon reasonable request to Belinda Reininger, belinda.m.reininger@uth.tmc.edu.
